# Measurement Noise Recommendation for Efficient Kalman Filtering over a Large Amount of Sensor Data

**DOI:** 10.3390/s19051168

**Published:** 2019-03-07

**Authors:** Sebin Park, Myeong-Seon Gil, Hyeonseung Im, Yang-Sae Moon

**Affiliations:** Department of Computer Science, Kangwon National University, Chuncheon-si, Gangwon-do 24341, Korea; sebinpark@kangwon.ac.kr (S.P.); gils@kangwon.ac.kr (M.-S.G.); ysmoon@kangwon.ac.kr (Y.-S.M.)

**Keywords:** kalman filtering, measurement noise, sensor data, filtering accuracy, wavelet transform, denoising autoencoder

## Abstract

To effectively maintain and analyze a large amount of real-time sensor data, one often uses a filtering technique that reflects characteristics of original data well. This paper proposes a novel method for recommending the *measurement noise* for Kalman filtering, which is one of the most representative filtering techniques. Kalman filtering corrects inaccurate values of input sensor data, and its filtering performance varies depending on the input noise parameters. In particular, if the noise parameters determined based on the user’s experience are incorrect, the accuracy of Kalman filtering may be reduced significantly. Based on this observation, this paper addresses how to determine the measurement noise variance, a major input parameter of Kalman filtering, by analyzing past sensor data and how to use the estimated noise to improve the filtering accuracy. More specifically, to estimate the measurement noise variance, two analytical methods are proposed: one a *transform-based* method using a *wavelet* transform and the other a *learning-based* method using a *denoising autoencoder*. Experimental results show that the proposed methods estimated the measurement noise variance accurately and were superior to the experience-based method in the filtering accuracy.

## 1. Introduction

Recent developments in sensor-based technologies such as 5G communications and IoT (Internet of Things) have increased innovative applications such as smart factories, smart cities, and wearable devices [1,2,3]. Since these smart applications generate a huge amount of sensor data, one needs an effective filtering process to extract meaningful information from those massive data [4,5]. Filtering is often used to refine the original data by removing unnecessary information or reducing the amount of data. Among various filtering techniques, this paper aims to improve the performance of Kalman filtering [6,7,8], which is widely used for noise reduction and correction of sensor data, for example, in the field of computer vision, signal processing, and robotics due to its nice optimality property under certain conditions.

Kalman filtering is an unsupervised filtering algorithm specialized to sensor data [9], which adjusts the currently measured sensor value by considering the past sensor data, for example, to reduce the noise in the measured value. Since it can provide values closer to the *actual sensor values* (without noise) than the simple measurement values, one often uses a Kalman filter to remove the noise of sensor data or to predict the next sensor values. Kalman filtering operates in two steps: prediction and update. First, in the prediction step, it estimates the next value to be measured from the current sensor based on the past sensor data. Next, in the update step, it refines the estimated value in the prediction step using the measured value at the present time, to obtain a value closer to the actual one.

This paper focuses on the input parameters used in Kalman filtering. Among many input parameters, the noise information of the sensor largely affects the filtering performance, and users normally determine the noise information based on their experience and knowledge on the sensor data. In particular, the *measurement noise (co)variance* is a representative parameter of the Kalman filter, and the expert usually estimates it from analyzing the complicated specification of sensing equipment. However, it is very difficult for ordinary users to understand accurate noise information included in the measurement process of the sensor. Thus, most users use an arbitrary value (mostly, close to 0) as the measurement noise variance, which is a major cause of a significant decrease in the accuracy of Kalman filtering. Moreover, these inaccurate predictions continue to accumulate and thus the user will continue to obtain inaccurate filtering results. To solve this problem, a novel method of estimating the measurement noise variance directly from input sensor data is proposed so that the user can effectively use Kalman filtering even when the sensor equipment do not provide any noise information.

For accurate estimation of the measurement noise variance, this paper proposes analytical methods analyzing past sensor data. In general, sensor data contain not only actual values but also the noise generated during the measurement process, and the noise value may vary by various factors such as individual sensors accuracy and the measurement environment. Obviously, if one can correctly estimate the measurement noise variance from the measured sensor values, they can obtain a more accurate filtering result than using an arbitrary value as the variance. To this end, this paper proposes *transform-based* and *learning-based* methods for estimating the measurement noise variance based on input sensor data.

First, the transform-based method uses *wavelet* transforms [10,11]. Briefly speaking, this approach applies the transform to sensor data and then uses the difference between the transformed data and the original sensor data as the measurement noise. Since a wavelet transform has the characteristics of removing the noise from sensor data, using this property, the noise included in the sensor data can inversely be estimated. A wavelet transform is widely used to analyze the global and regional trends of sequence data by extracting frequency characteristics of the data. In this paper, the Daubechies wavelet [12] is used as it is one of the most commonly used wavelets.

Second, the learning-based method uses *denoising autoencoders* [13,14,15]. A denoising autoencoder is an artificial neural network that can estimate the original data from the measured data containing noise. Thus, by using a denoising autoencoder, the original data and the noise data can be separated from the measured data, and, as a result, the noise data can be used as the measurement noise. In this paper, a denoising autoencoder is constructed with multiple convolution layers to improve the estimation accuracy of the measurement noise variance.

Extensive experiments on real datasets showed that the proposed analytical methods significantly outperformed the arbitrary noise variance selection method. First, in the experiment on Kalman filtering accuracy, our recommendation-based methods performed filtering up to 2.5 times more accurately than the arbitrary noise variance selection method. Next, in the experiment on estimation accuracy of the measurement noise variance, the proposed methods estimated the measurement noise variance accurately. Based on these experimental results, it is concluded that the proposed analytical methods are useful not only for ordinary users but also for experts to easily determine the measurement noise variance of Kalman filtering and improve the filtering accuracy of sensor data.

The main contributions of the paper are summarized as follows:

- To estimate the measurement noise variance accurately, two analytical methods are proposed: one a transform-based method using a wavelet transform and the other a learning-based method using a denoising autoencoder.
- A practical approach for Kalman filtering for streaming data is presented, which exploits the recommended measurement noise variance by the proposed methods.
- Through extensive experiments on real datasets, the effectiveness of the proposed methods was validated, and it was shown that they estimated the measurement noise variance accurately.

The rest of the paper is organized as follows. Section 2 describes the related work on Kalman filtering, wavelet transforms, and denoising autoencoders. It also reviews the previous work on recommending parameters in Kalman filtering. Section 3 discusses the difficulty in using Kalman filtering and proposes an analytical approach of using the recommended measurement noise variance as a solution. Section 4 formally explains our analytical methods of estimating the measurement noise variance. Section 5 provides the experimental results to show the superiority of the proposed methods. Finally, Section 6 concludes and discusses future work.

## 2. Related Work

### 2.1. Kalman Filtering

Kalman filtering [6] is used to correct noisy time-series data [16,17] by expressing the data as a state space model and applying probabilistic estimation to the model. It is often used to remove the noise from sensor data or estimate the next sensor values. For example, one can use Kalman filtering in a power measurement process to remove high noise in raw voltage data. It can also be used to estimate non-measured measurements from other measured measurements, for instance, to estimate the velocity from location measurements of a navigation system. In particular, when the Kalman filter is used for noise reduction, it aims to minimize the error between the actual value having no noise and the corrected value. Since Kalman filtering uses past data recursively, one can expect more accurate results than a filtering method based only on incoming measurements. In addition, as it processes new measurement data quickly, it is suitable for handling continuously generated sensor data.

To use Kalman filtering, one needs to build state equations such as in Equation (1) where the true state $x_{t}$ at time *t* is assumed to be derived from the previous state $x_{t-1}$ according to $x_{t}=F_{t}x_{t-1}+w_{t}$, and a measurement $z_{t}$ of the true state $x_{t}$ is assumed to be made according to $z_{t}=H_{t}x_{t}+v_{t}$. Then, Kalman filtering estimates the true state in two steps. First, it predicts the (a priori) state estimate ${\hat{x}}_{t}^{-}$ from the previous state estimate ${\hat{x}}_{t-1}$, and next it updates ${\hat{x}}_{t}^{-}$ by using the current measurement $z_{t}$ to obtain the (a posteriori) state estimate ${\hat{x}}_{t}$ as shown in Equation (2). For more details on the working mechanism, equations, and parameters of Kalman filtering, the reader is referred to [6,18,19,20].

(1) $$\left\{\begin{array}{c} x_{t}=F_{t}x_{t-1}+w_{t}\\ z_{t}=H_{t}x_{t}+v_{t} \end{array}\right.,\;\mathrm{where}\;\left\{\begin{array}{c} x_{t}:\mathrm{true}\;\mathrm{state}\;\mathrm{at}\;\mathrm{time}\;t\;(a\;\mathrm{vector}\;\mathrm{of}\;\mathrm{real}\;\mathrm{numbers})\\ z_{t}:\mathrm{measurement}\;\mathrm{at}\;\mathrm{time}\;t\;(\mathrm{or}\;\mathrm{observation})\\ F_{t}:\mathrm{state}\;\mathrm{transition}\;\mathrm{model}\;\mathrm{applied}\;\mathrm{to}\;x_{t-1}\\ H_{t}:\mathrm{observation}\;\mathrm{model}\\ Q_{t}:\mathrm{process}\;\mathrm{noise}\;(\mathrm{co})\mathrm{variance},\;w_{t}∼N\left(0,Q_{t}\right)\\ R_{t}:\mathrm{measurement}\;\mathrm{noise}\;(\mathrm{co})\mathrm{variance},\;v_{t}∼N\left(0,R_{t}\right) \end{array}\right.$$

(2) $$Predict:\left\{\begin{array}{c} {\hat{x}}_{t}^{-}=F_{t}{\hat{x}}_{t-1}\\ P_{t}^{-}=F_{t}P_{t-1}F_{t}^{T}+Q_{t} \end{array}\right.,\;\;Correct:\left\{\begin{array}{c} K_{t}=P_{t}^{-}H_{t}^{T}{\left(H_{t}P_{t}^{-}H_{t}^{T}+R_{t}\right)}^{-1}\\ {\hat{x}}_{t}={\hat{x}}_{t}^{-}+K_{t}\left(z_{t}-H_{t}{\hat{x}}_{t}^{-}\right)\\ P_{t}=\left(I-K_{t}H_{t}\right)P_{t}^{-}{\left(I-K_{t}H_{t}\right)}^{T}+K_{t}R_{t}K_{t}^{T} \end{array}\right.$$

To use Kalman filtering correctly, the user needs to input detailed information about the sensor data. In particular, the process noise (co)variance *Q* and the measurement noise (co)variance *R* in Equation (1) are important parameters for determining the performance of the Kalman filter. The process noise can be thought of as being generated by the external environment such as ultraviolet rays and wind, and the domain expert may determine the value, but usually one uses a value close to zero. In contrast, the measurement noise comes from the measuring device, and it is difficult for an ordinary user, who has no or little knowledge about the device, to obtain the correct value of the measurement noise variance *R*. If *R* is unknown, the user simply uses an experience-based arbitrary value, which might significantly degrade the filtering accuracy. To solve this problem, in this paper, an analytical method is proposed, which estimates the measurement noise variance *R* by analyzing past sensor data and recommends the estimated value to the user when *R* is not given.

There have been a number of research efforts on estimating the parameter values of Kalman filtering. Mehra [21] estimated the process noise covariance *Q* and the measurement noise covariance *R* by using the innovation property of an optimal filter [22] and by iteratively solving a set of equations. Godbole [23] extended the result of Mehra’s work [21] to the case where the means of *Q* and *R* are unknown. Lee [24] proposed a more direct approach based on a correlation method to reduce the computational complexity. Bulut et al. [25] reviewed and compared the classical innovations and output covariance techniques to estimate *Q* and *R* from the measured data. Although similar in spirit to our work in that they also estimate noise information from past data, all these approaches require long computation time due to a large number of equations to solve. Moreover, they are not applicable if the number of unknown variables is greater than the number of equations required for estimation. Thus, it is difficult for ordinary users to use these methods practically. In contrast, our proposed methods depend only on the available sensor data and can be applied even to a small amount of data.

Several Bayesian approaches have also been proposed for adaptive Kalman filtering [26]. To name a few, Särkkä and Nummenmaa [27] proposed a recursive algorithm for estimating the time-varying measurement noise covariance based on variational Bayesian methods [28]. Their method, however, assumes an accurate process noise covariance and its performance will degrade if it uses an inaccurate process noise covariance. Recently, Huang et al. [29] and Sun et al. [30] proposed a variational Bayesian adaptive Kalman filter with inaccurate process and measurement noise covariances, where the time-varying measurement noise covariance can be inferred by choosing inverse Wishart priors. All these variational Bayesian approaches involve fixed point iterations to infer the measurement noise covariance at each time step. In contrast, in this paper, a simpler approach is taken based on wavelet transforms and denoising autoencoders, which can estimate the time-varying measurement noise variance in real time without fixed point iterations.

### 2.2. Wavelet Transforms

In this paper, as a transform-based method for estimating the measurement noise variance, a wavelet transform is used. A wavelet transform is widely used in various signal processing applications including noise removal, image compression, and sound compression. It converts a signal from a time-series domain into a frequency domain and uses the extracted frequency components instead of time-series data. For example, the integral wavelet transform of f(t), given a basis function ψ with a scale parameter *s* and a translating parameter *u*, is defined as follows [31]:

(3) $$W_{f}\left(u,s\right)=\int_{-\infty }^{\infty }f\left(t\right)\;\frac{1}{\sqrt{s}}\;\psi^{*}\left(\frac{t-u}{s}\right)dt$$

where * in $\psi^{*}$ denotes the complex conjugate operator. As a basis function of the wavelet transform, sine and cosine functions as well as Haar and Daubechies can be used. The result of the wavelet transform is largely divided into a high frequency part and a low frequency part, where the high frequency part represents the details about the input data such as abrupt changes or discontinuities, and the low frequency part represents the coarse structure or the approximation of the input data. By exploiting the high frequency part from the transform result in estimating the measurement noise variance, the filtering accuracy of the Kalman filter can be improved. For a detailed description of wavelet transforms, the reader is referred to [31].

**Figure 1:**
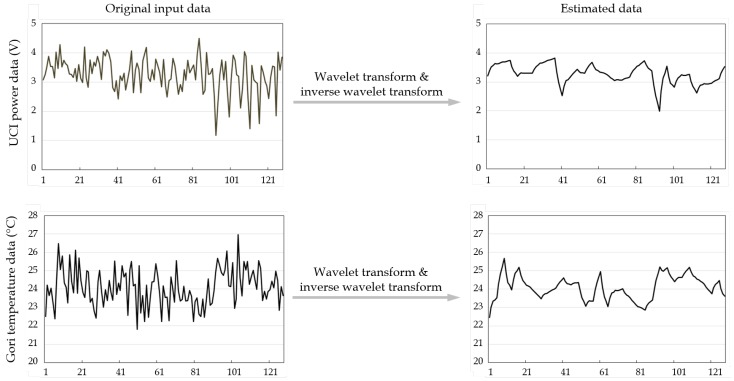
An example of applying wavelet transforms to sensor data.

Figure 1 shows an example of applying wavelet transforms to sensor data. The first column of the figure shows two raw datasets that contain noise data: (1) power data of UCI (University of California, Irvine) repository [32]; and (2) temperature data of Gori nuclear plant [33]. The second column shows the results of estimating the original data by applying wavelet transforms to the raw noisy data. As shown in the figure, the wavelet transforms generate the original data by sufficiently reflecting the tendency of the input data and at the same time by removing the noise from the raw data. Therefore, in this paper, wavelet transforms are used, which are simple and efficient, for estimating the measurement noise variance for Kalman filtering.

### 2.3. Denoising Autoencoders

**Figure 2:**
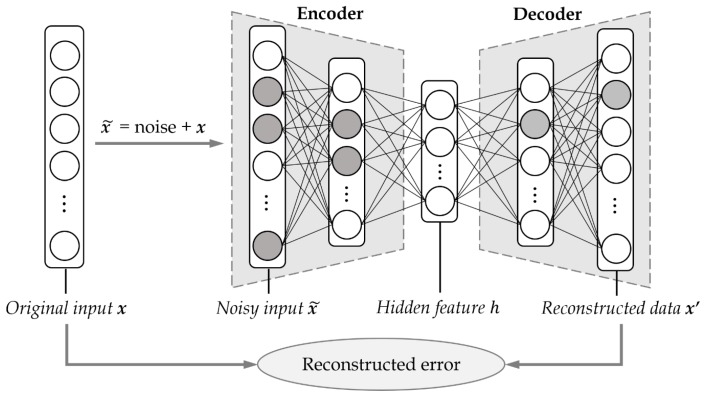
The overall structure of a denoising autoencoder.

Our learning-based approach uses denoising autoencoders [13], which are often used in image and signal processing and data preprocessing. A denoising autoencoder is a kind of autoencoders [34,35], which is an artificial neural network that can estimate the original data by removing the noise from noisy data. A conventional autoencoder is a neural network that generates an output from an input over the corresponding model, and it consists of an encoder and a decoder. In general, the encoder gets the data *x* and learns its features by dimensionality reduction, and the decoder tries to restore the data *x* from the encoded features. As shown in Figure 2, a denoising autoencoder maintains an encoder–decoder structure as in the conventional autoencoder, but it uses corrupted data as input for the encoder, while the loss of the model is computed using the original data without noise. This method basically assumes that the characteristics representing the input data can be reliably extracted even in the presence of noise [13].

The working mechanism shown in Figure 2 is as follows. First, noise is added to the original input *x* to create a noisy input $\tilde{x}$. Then, the generated noisy input $\tilde{x}$ is provided to a neural network model and the encoding–decoding process is performed. Equation (4) is an encoding expression in which the input data $\tilde{x}$ is mapped to the hidden feature *h* through the encoder, and Equation (5) is a decoding expression in which the data $x^{\prime }$ is reconstructed from the feature *h* through the decoder. (In general, Sigmoid() is used as an activation function in autoencoders. In this paper, however, LeakyReLU() is used instead of Sigmoid() for better performance. See Section 5.1 for details.) In θ={W,b} and $\theta^{\prime }=\left\{W^{\prime },b^{\prime }\right\}$ of Equations (4) and (5), *W* and $W^{\prime }$ represent weights, while *b* and $b^{\prime }$ represent biases.

(4) $$\begin{array}{cc} h & =f_{\theta }\left(\tilde{x}\right)=Sigmoid\left(W\tilde{x}+b\right) \end{array}$$

(5) $$\begin{array}{cc} x^{\prime } & =g_{\theta^{\prime }}\left(h\right)=Sigmoid\left(W^{\prime }h+b^{\prime }\right) \end{array}$$

In general, the loss function L() of the denoising autoencoder model is set to the squared error between the reconstructed data $x^{\prime }$ and the original data *x*, as shown in Equation (6), which is usually averaged over some input training set. Equation (6) corresponds to the reconstructed error in Figure 2. Then, the model is optimized so that the loss function can be minimized, as in Equation (7), where $x^{\left(i\right)}$ and $x^{\prime }{}^{\left(i\right)}$ denote the *i*th elements of *x* and $x^{\prime }$, respectively.

(6) $$\begin{array}{cc} L(x,x^{\prime }) & =||x-x^{\prime }{\left|\right|}^{2} \end{array}$$

(7) $$\begin{array}{cc} \theta *,\theta^{\prime }* & =\underset{\theta ,\theta^{\prime }}{\arg\;\min}\;\frac{1}{n}\;\sum_{i=1}^{n}L\left(x^{\left(i\right)},x^{\prime }{}^{\left(i\right)}\right) \end{array}$$

In this paper, the denoising autoencoder model is trained to estimate the original input data under various noise settings, which is then used to compute the measurement noise variance for Kalman filtering.

## 3. Kalman Filtering Using the Recommended Measurement Noise Variance

In general, the input data to Kalman filtering contains various noise depending on the performance of the measuring device, and thus there is a difference between the input data and the original data. That is, Kalman filtering receives the sensor data, which are a mixture of the original data and the device noise. To accurately filter the noise with a Kalman filter, one needs to know the variance of the added noise in the measurement process, which is called the measurement noise variance *R*. If the measuring device itself provides noise information, the user may use the information for Kalman filtering. In actual streaming environments, however, it is very difficult for ordinary users to obtain this noise information from the complicated device configuration, in which case they need to determine the measurement noise variance based on their experience. If the variance *R* chosen based on the user’s experience is close to the actual noise variance of the sensor device, then the filtering accuracy might be relatively high, but otherwise the accuracy would be greatly degraded. As a result, to obtain more accurate filtering results, it is better to use an analytical method that estimates the noise variance directly from the sensor data rather than using an experience-based arbitrary method.

**Figure 3:**
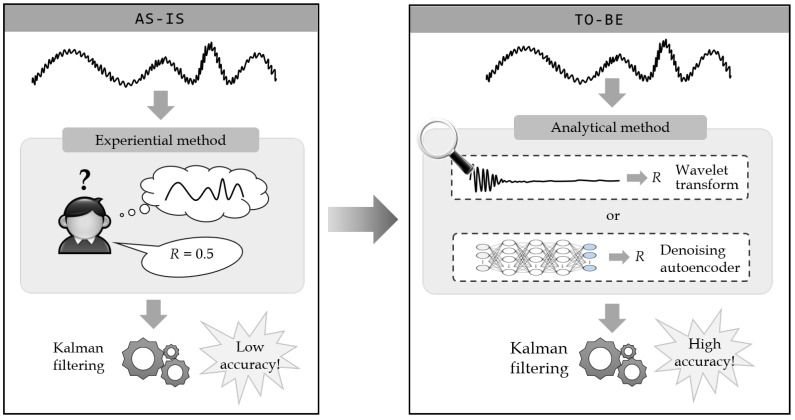
Decision process of the measurement noise variance *R* in existing and proposed methods.

Figure 3 shows how the measurement noise variance is determined in the conventional Kalman filtering and the proposed framework. The AS-IS in the figure represents the experiential method where the user determines the measurement noise variance *R* based on the experience. Due to lack of device noise information, the user experientially determines the noise parameter by investigating the measured sensor data. In contrast, the TO-BE in the figure represents the proposed analytical method that recommends the measurement noise variance *R* by analyzing the sensor data. The measurement noise variance is the variance of the noise contained in the input data, and the analytical method obtains the variance by comparing the actual sensor data containing the noise and the estimated data from which the noise is removed. Thus, in the analytical method, one important issue is how to estimate the original data from the noisy sensor data. For this, transform-based and learning-based methods are proposed. First, the transform-based method uses wavelet transforms, which are widely used for removing the noise from numeric sequence data, and using these transforms the original data can be estimated by removing the noise from the sensor data. Second, the learning-based method uses denoising autoencoders, which are also used for removing the noise from the sequence data, and the original data can be estimated by training a neural network model over the sensor data.

Algorithm 1 shows a generic structure of Kalman filtering using the proposed analytical method. It takes as input a data stream *S* which is partitioned into a set of data sequences $T_{1},T_{2},\ldots ,T_{n}$ where each sequence $T_{i}$ is of size *w*. For each step, the $T_{i}$ sequence is used to estimate the measurement noise variance *R* which is then used in Kalman filtering of the next $T_{i+1}$ sequence. By computing the measurement noise variance from $T_{i}$ and applying the Kalman filter to the same $T_{i}$ using the previously computed variance in parallel, Algorithm 1 can easily be applied in streaming environments with a computational cost bounded only by the complexity of the Kalman filter. Specifically, on Line (2), the first *w* entries of the input *S* are stored into $S^{\prime }$ as it is, since the measurement noise variance is not estimated yet. On Line (4), the current *w* entries of the input *S* are exploited to determine the measurement noise variance *R* by applying a given analytical function $f_{R}\left(\right)$. As mentioned above, either a wavelet transform or a denoising autoencoder can be used as the function $f_{R}\left(\right)$. On Line (6), the Kalman filter is applied to the next *w* entries using the recommended variance *R* and the result is stored into $S^{\prime }$. The steps on Lines (4)–(6) are repeated until the end of the input is reached. Finally, on Line (8), the newly constructed sequence $S^{\prime }$ is returned as the filtered sequence.

**Algorithm 1** Kalman filtering using the recommended measurement noise variance.**Input:**      S[1:length]: a numeric sequence of sensor data;      $f_{R}\left(\right)$: a function for a measurement noise variance recommendation;      *w*: a window size used for computing the measurement noise variance; **Output:**      $S^{\prime }\left[1:length\right]$: a filtered sequence by the Kalman Filter; **begin**      (1) i:=1      (2) $S^{\prime }\left[1:w\right]:=S\left[1:w\right]$;                   // Use the first *w* entries of *S* for $S^{\prime }$ as it is.      (3) **while** i+w−1≤length **do**      (4)       $R:=f_{R}\left(S\left[i:i+w-1\right]\right)$;     // Determine *R* by using the *w* entries.      (5)       i:=i+w;                              // Increase *i* by *w*.      (6)       $S^{\prime }\left[i:i+w-1\right]:=$ KalmanFilter(S[i:i+w−1],R);                 // Adjust the next *w* entries by applying *R* to the Kalman filter.      (7) **end**      (8) **return**
$S^{\prime }\left[1:length\right]$; **end**

## 4. Measurement Noise Variance Recommendation Methods

This section describes in detail our measurement noise variance recommendation methods. The measurement noise variance recommendation mainly consists of two steps: the original data estimation step and the measurement noise variance calculation step. In particular, in the original data estimation step, a transform-based method and a learning-based method are proposed.

### 4.1. Wavelet Transform-Based Measurement Noise Variance

**Figure 4:**
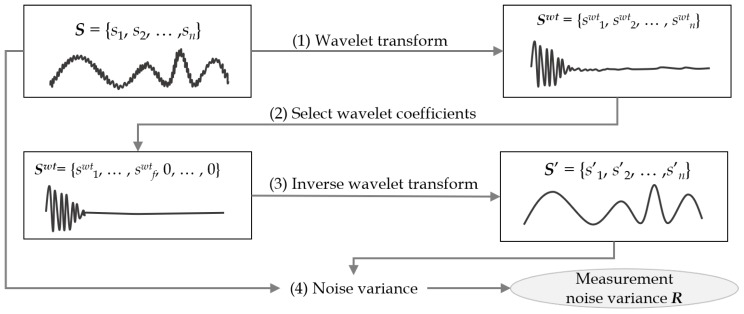
Measurement noise variance estimation using a wavelet transform.

Figure 4 illustrates the steps of estimating *R* in the wavelet transform-based method. It consists of four steps: (1) applying a wavelet transform; (2) choosing a few energy-concentrated coefficients; (3) performing the inverse wavelet transform; and (4) calculating the measurement noise variance *R* from the sensor and recovered data. More specifically, in Step (1), it applies a wavelet transform to the input sensor data *S* of length *n*. The wavelet-transformed sequence $S^{wt}$ contains wavelet coefficients having frequency information of the input sequence *S*. In Step (2), it takes the first f(≪n) coefficients only and sets the remaining coefficients to 0. In Step (3), it estimates the noise-removed original sequence $S^{\prime }$ by applying the inverse wavelet transform to the high frequency sequence $S^{wt}$. Finally, in Step (4), it determines *R* by calculating the variance between the input sequence *S* and the recovered sequence $S^{\prime }$. A discussion of the accuracy of the noise estimated in this way can be found in [10].

Algorithm 2 shows the pseudocode of Figure 4. The inputs are sensor data $S_{sensor}$ and a number *f* of wavelet coefficients to be selected, and the output is *R*. On Line (1), it applies a wavelet transform to $S_{sensor}$ and obtains $S^{wt}$ having frequency coefficients. On Line (2), it discards low energy coefficients by setting them to 0 and takes only high energy coefficients. On Line (3), it estimates the original sequence $S_{estimate}$ by applying the inverse wavelet transform to $S^{wt}$. Since noise coefficients from $S^{wt}$ are removed, $S_{estimate}$ is expected to have less noise. On Line (4), it calculates *R* from $S_{sensor}$ and $S_{estimate}$, and finally on Line (5), it returns *R* as the measurement noise variance.

**Algorithm 2**$f_{WT}\left(\right)$: Measurement noise variance by wavelet transform.**Input:**      $S_{sensor}$: a numeric sequence of sensor data, ($n=|S_{sensor}|$);      *f*: the number of wavelet coefficients to be selected; **Output:**      *R*: measurement noise variance of $S_{sensor}$; **begin**      (1) $S^{wt}:=$ WaveletTransform$\left(S_{sensor}\right)$;      (2) $S^{wt}:=$ Concatenate$(S^{wt}\left[1:f\right],$Array.zeros(n−f));     // Take high energy coefficients.      (3) $S_{estimate}:=$ InverseWaveletTransform$\left(S^{wt}\right)$;      (4) R:= CalculateVAR$\left(S_{sensor},S_{estimate}\right)$;     // Calculate the variance between two sequences.      (5) **return**
*R*; **end**


### 4.2. Denoising Autoencoder-Based Measurement Noise Variance

**Figure 5:**
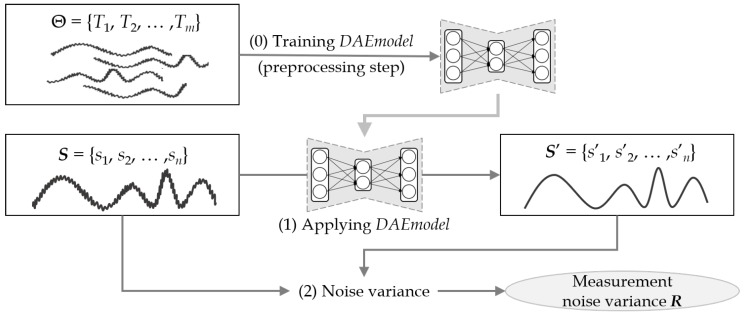
Measurement noise variance estimation using a denoising autoencoder.

Figure 5 illustrates the steps of the denoising autoencoder-based method. Unlike the previous transform-based method, this learning-based method first performs a preprocessing step to train a denoising autoencoder model, which is called *DAEmodel* in this paper. As shown in the figure, the method consists of three steps: (0) training *DAEmodel* in the preprocessing step; (1) estimating the original sequence by using the trained *DAEmodel*; and (3) calculating the measurement noise variance *R*. More specifically, in Step (0), it trains *DAEmodel* using a given training set Θ of sensor data $T_{i}$. Note that this preprocessing step is performed just once. In Step (1), it estimates the original sequence $S^{\prime }$ by applying the input sensor data *S* to *DAEmodel*. In Step (2), it calculates the measurement noise variance *R* from two sequences *S* and $S^{\prime }$. In this learning-based method, how the denoising autoencoder is configured is important, and the model configuration is discussed in detail in Section 5.1.

Algorithm 3 shows the pseudocode of Figure 5. The inputs are a training set Θ and an input sensor data $S_{sensor}$, and the output is *R*. It is assumed that the training set Θ consists of *m* sequences $T_{1},T_{2},\ldots ,T_{m}$, which are collected from previously accumulated sensor data. Unlike Algorithm 2, Algorithm 3 does not require additional input parameters such as *f*, but it instead requires a training set in the preprocessing step. In practice, to build a training set from noise-corrupted sensor data, one may exploit autoencoders or other unsupervised denoising techniques in the preprocessing step. (Although the training data obtained by unsupervised denoising techniques are not necessarily the same as the original data, a denoising autoencoder can still be trained to produce a high-level representation that is robust to various noise and able to estimate the original data fairly accurately.) In Step (0), it obtains a denoising autoencoder model, *DAEmodel*, using the input training set Θ. Once this training step is completed, *DAEmodel* plays a role of removing noise from the input sequence data, and it can repeatedly be used in the next subsequent steps. In Step (1), it applies the input sensor data $S_{sensor}$ to the trained *DAEmodel* and estimates the noise-removed original sequence $S_{estimate}$. In Steps (2) and (3), the variance is computed and returned as *R*.

**Algorithm 3**$f_{DAE}\left(\right)$: Measurement noise variance by denoising autoencoder.**Input:**      $\Theta =\{T_{1},T_{2},\ldots ,T_{m}\}$: a training set of *m* sensor data;      $S_{sensor}$: a numeric sequence of input sensor data; **Output:**      *R*: measurement noise variance of $S_{sensor}$; **begin**      (0) *DAEmodel*
:= TrainModel(Θ);            // Build a denoising autoencoder model in the preprocessing step.      (1) $S_{estimate}:=$ ApplyModel$\left(S_{sensor},\mathrm{DAEmodel}\right)$;      (2) R:= CalculateVAR$\left(S_{sensor},S_{estimate}\right)$;     // Calculate the variance between two sequences.      (3) **return**
*R*; **end**


## 5. Experimental Evaluation

This section experimentally evaluates the filtering and estimation accuracy of the proposed methods. Section 5.1 explains experimental data and parameter settings. Then, the experimental results are presented in Section 5.2 and Section 5.3.

### 5.1. Experimental Setup

In the experiment, two accuracy measures were evaluated: (1) Kalman filtering accuracy; and (2) noise variance estimation accuracy. First, the Kalman filtering accuracy experiment compared the existing experiential Kalman filtering and the proposed analytical approach. Next, the noise variance estimation accuracy experiment compared the actual noise variance of the sensor data and the measurement noise variance estimated by each of the proposed methods.

**Figure 6:**
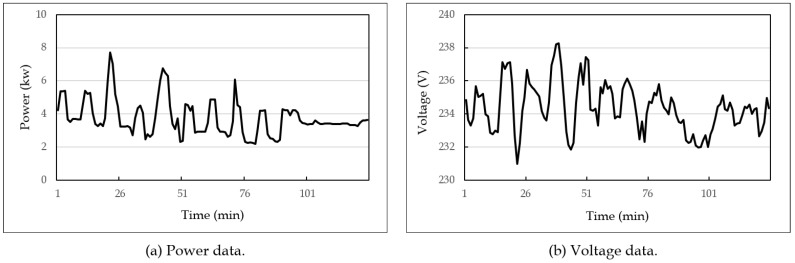
An example part of power consumption data used in the experiment.

The hardware platform was a workstation equipped with Intel^®^ Core${}^{\mathrm{TM}}$ i5-2400 CPU 3.10 GHz, 16 GB RAM, and 128 GB SSD. For the learning process of the denoising autoencoder method, GPU of NVIDIA GeForce GTX 970 with 4 GB was used. The software platform was the Windows 10 operating system, and the development and execution environment was IntelliJ IDEA and Anaconda 4.4.8. As the experimental data, household power consumption data were used, which were obtained from the UCI machine learning repository [32]. The power consumption data consist of 2,075,259 measurement values collected every minute from December 2006 to November 2010 for 47 months. Figure 6 shows an example part of the experimental data: Figure 6a for power data and Figure 6b for voltage data.

In the experiment, these power and voltage data, such as in Figure 6, were regarded as original data. The input sensor data to the Kalman filter were then obtained by adding Gaussian white noise to these original data, since it is a good approximation of many real-world environments. More precisely, the noise data were generated from the original data using the normal distribution with the standard deviation σ×n/100 for various *n* where σ is the standard deviation of the original data, as shown in Equation (8).

(8) $$\left\{\begin{array}{c} O∼N\left(mean,\sigma^{2}\right)\\ X∼N(0,\left({\sigma \times n/100)}^{2}\right) \end{array}\right.,\;\mathrm{where}\;\left\{\begin{array}{c} O:\mathrm{original}\;\mathrm{data}\\ \sigma :\mathrm{standard}\;\mathrm{deviation}\;\mathrm{of}\;O\\ X:\mathrm{noise}\;\mathrm{data}\;\mathrm{to}\;\mathrm{be}\;\mathrm{added}\;\mathrm{to}\;O\\ \sigma \times n/100:\mathrm{standard}\;\mathrm{deviation}\;\mathrm{of}\;X \end{array}\right.$$

The proposed measurement noise variance recommendation methods require sequence data of a certain length. Thus, the sensor data were divided into sequences of lengths 512, 1024, and 2048, and these three types of sequences were used in the experiment. More specifically, given a stream $T_{1},T_{2},\ldots ,T_{n}$ of sequence data, the $T_{i}$ sequence data were used to estimate the measurement noise variance *R* using the proposed methods, and then *R* was exploited when the Kalman filter was applied to the $T_{i+1}$ sequence data. The wavelet transform-based method used FWT (fast wavelet transform) [36] as the wavelet function and Daubechies-4 [37] as the basis function since they are the most commonly used wavelets. Other wavelets can also be used. The number *f* of wavelet coefficients was set to 128, 256, and 512, i.e., 1/4 of the sequence lengths, respectively.

**Figure 7:**
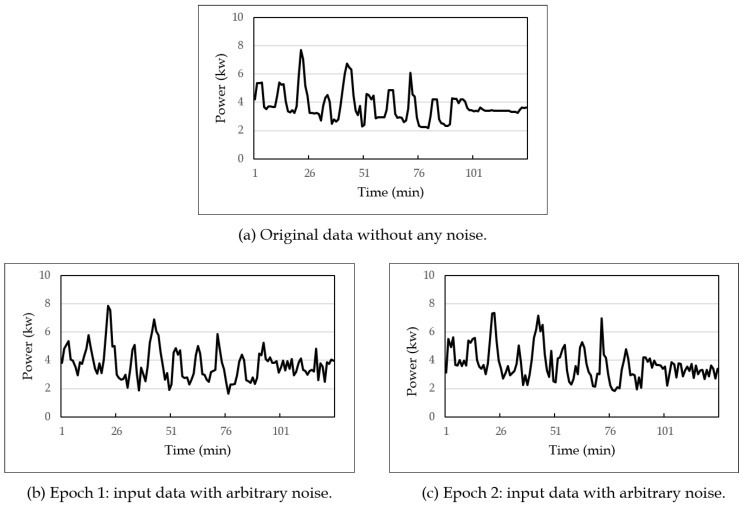
An example of training data used for each learning epoch.

In the denoising autoencoder-based method, first, *DAEmodel* was built using a training set. For this, 12,000 sequences of lengths 512, 1024, and 2048 were constructed from the original data, which were then divided into training data of 7200 sequences, validation data of 2400 sequences, and test data of 2400 sequences. The training process needs to input noise-added sequences into *DAEmodel* rather than original sequences. Thus, for each learning iteration, input sequences were generated by adding various noise values with arbitrary standard deviations to the original sequences. Then, *DAEmodel* was trained using these noise-added input sequences. Figure 7 shows an example of original and input sequences: Figure 7a shows an original sequence before adding noise, and Figure 7b,c shows different input sequences provided to *DAEmodel*. As shown in the figure, input sequences for each epoch were changed, and accordingly, various noise values could be reflected to *DAEmodel*.

**Figure 8:**
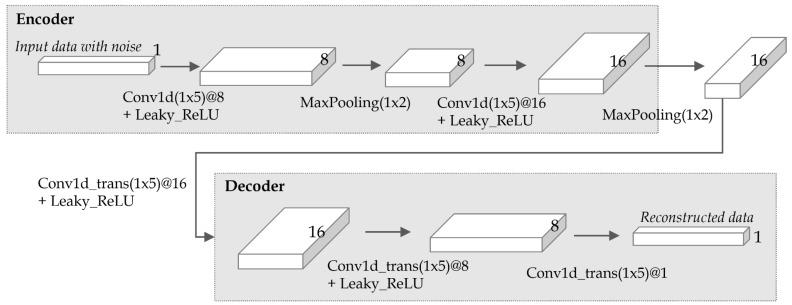
The detailed structure of the denoising autoencoder used in the experiment.

Figure 8 shows the structure of the denoising autoencoder used in the experiment. As shown in the figure, the model had a symmetric structure of an encoder and a decoder, where the encoder consisted of two convolution layers and two pooling layers, and the decoder consisted of three transpose-convolution layers. Each convolution layer in the encoder used 1×5 filters to extract various feature vectors, and each transpose-convolution layer in the decoder also used 1×5 filters to reduce and summarize feature vectors. As an activation function between layers, LeakyReLU() (leaky rectified linear unit) [38] was used. Besides LeakyLeRU, other activation functions such as Sigmod() and tanh() were tested, but LeakyReLU() showed the best result in terms of filtering accuracy. Table 1 shows the hyperparameters used for training the model. As shown in the table, the learning rate was set to 0.01, the batch size to 200, and the optimizer to Adam. The number of epochs was set to 500 since the loss, the difference between original and reconstructed data, converged at around 500 epochs.

With this setup, one complete naïve training for power data using fixed values of parameters took about 6.75 h when only CPU was used and about 4.5 h when GPU was used (similarly for voltage data). During the training, the best *DAEmodel*, which gave the best performance for validation data, was chosen and used in the subsequent experiments. The result on measurement noise variance estimation presented in Section 5.3 can be thought of as the result of applying *DAEmodel* to test data. The result of applying *DAEmodel* to validation data is not presented since it was used in choosing the best model and thus the result for validation data is better than the result for test data, which are unseen by the model during training.

### 5.2. Accuracy Evaluation of Kalman Filtering

This section presents the filtering accuracy of the experiential and analytical methods. Specifically, the Kalman filter was applied to power and voltage data using the measurement noise variance estimated by the proposed methods. For each method, the Euclidean distance [16,39] between the original data and the filtered data was calculated and regarded as the filtering accuracy of the method. For the experiential method, the measurement noise variance *R* for each sequence of data was set to an arbitrary value between 0 and 30×σ, where σ is the standard deviation of the original data.

**Figure 9:**
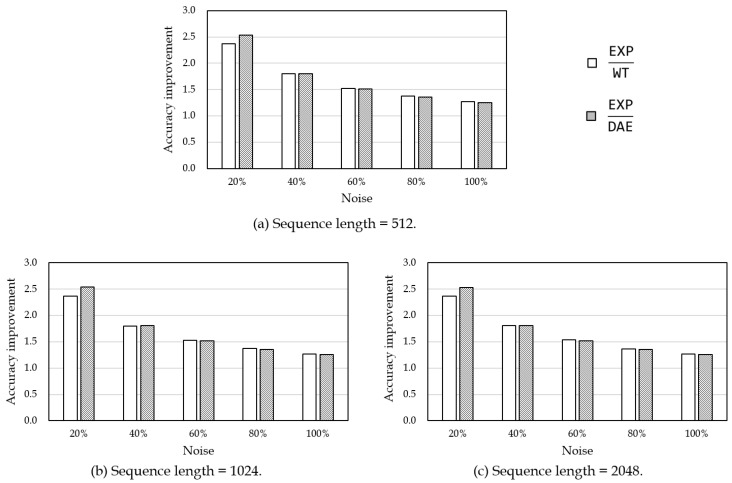
Filtering accuracy improvement on power data of different sequence lengths.

Table 2 and Figure 9 show the experimental result on power data. Table 2 shows the filtering accuracy of each method for different sequence lengths, and Figure 9 shows the filtering accuracy improvement rate of the proposed analytical methods compared to the experiential method. In the experiment, input data were generated from the original data by adding noise with a standard deviation of 20%, 40%, 60%, 80%, and 100% of the standard deviation of the original data, respectively, using Equation (8). The same experiment was repeated 10 times and their average was used as the result. In particular, the result of the experiential method can be thought of as an average performance of choosing an arbitrary value for the measurement noise variance. In Table 2 and Figure 9, EXP, WT, and DAE denote Kalman filtering using the experiential method, the wavelet transform-based method, and the denoising autoencoder-based method, respectively.

As shown in Table 2, the Euclidean distances of the proposed methods WT and DAE were much shorter than those of the experiential method EXP. This means that the analytical methods performed more accurate noise filtering than the experiential method. In Figure 9, the proposed methods of analytically estimating the measurement noise variance improved the filtering performance up to 2.5 times higher than the experiential method of using arbitrary noise variance. In particular, DAE was the best when the noise was added with a standard deviation of 20–40% of the standard deviation σ of the original data, and WT was the best when the noise was added with a standard deviation of above 60% of σ. This was partly because the denoising autoencoder was trained well for low noise variance cases, while it was not for high noise variance cases. To solve this problem, more layers could be used in the neural network model or different activation functions could be tried. However, since WT already showed sufficiently high performance, further optimization for DAE is left as a future study.

**Figure 10:**
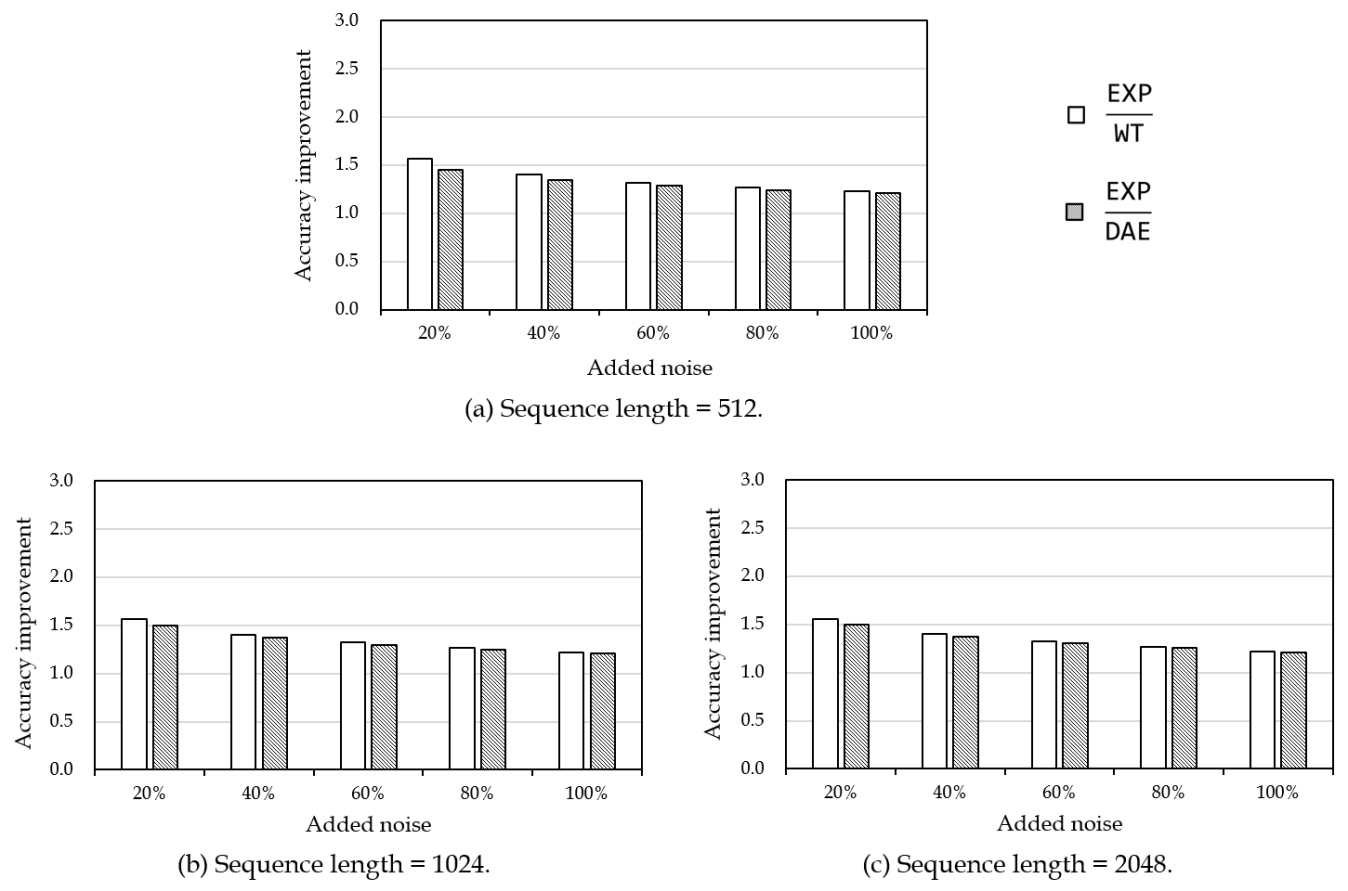
Filtering accuracy improvement on voltage data for different sequence lengths. The boldface numbers denote the best value in each experiment.

Table 3 and Figure 10 show the filtering accuracy result on voltage data. Similar to the power data, WT and DAE outperformed EXP. As shown in Figure 10, WT and DAE improved filtering performance up to 1.5 times higher than EXP. In particular, WT showed better performance than DAE in all cases. This was because WT was not much influenced by absolute values of noises, while the neural network of DAE was not well trained because of the large noise values. Specifically, the average of voltage data was about 241, which was much larger than 1.09, the average of power data. As explained above, the performance of DAE might be improved by using more layers and different activation functions.

**Figure 11:**
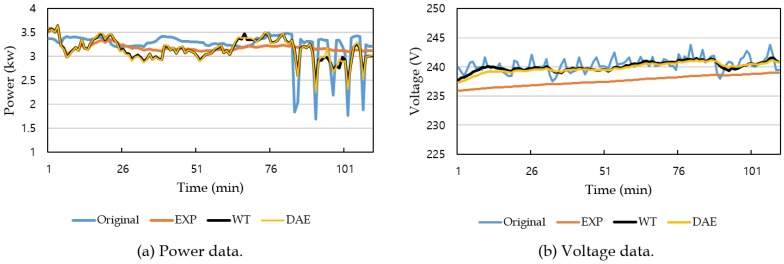
Example results of Kalman filtering.

Figure 11 shows example results of applying the Kalman filter to power and voltage data. In this figure, the input power (respectively, voltage) data to the Kalman filter were obtained by adding noise to the original power (respectively, voltage) data with a standard deviation of 60% (respectively, 20%) of that of the original data. As with the aforementioned experimental results, the Kalman filter produced a better result when using the measurement noise variance estimated by either the wavelet transform-based or the denoising autoencoder-based method than the experiential method, and the filtering performances of WT and DAE were comparable to each other.

Table 4 shows the average computation time of WT and DAE taken to estimate the measurement noise variance for power and voltage data. Note that the unit is millisecond. As shown in the table, both WT and DAE computed the measurement noise variance instantly, although DAE was slightly faster than WT. For example, after 512 data were accumulated, the wavelet transform (or the denoising autoencoder) was invoked and the computation of the measurement noise variance for power data was finished in 9.32 ms (or 2.14 ms) on average. This result implies that the performance of our methods depended only on the performance of Kalman filtering. Moreover, although both power and voltage data were sampled every minute, the sampling frequency was not relevant to the performance of the proposed methods. Indeed, even if data were sampled every second or every 100 ms, our methods would still work well. That is, the proposed methods could also be applied to other typical signals such as a sinusoidal signal, with a similar performance result.

### 5.3. Accuracy Evaluation of Measurement Noise Variance Estimation

The proposed methods were evaluated for how accurately they estimated the measurement noise variance *R*. The data and parameters used in the experiment were the same as presented in Section 5.2. Table 5 shows the experimental results obtained by estimating *R* with the proposed methods in the power data and comparing it with the actual noise variance. In the table, $R_{real}$ means the variance of the actual noise embedded in the original power data, and $R_{WT}$ and $R_{DAE}$ are the measurement noise variance estimated by WT and DAE, respectively. The experiments compared how similar $R_{WT}$ and $R_{DAE}$ were to $R_{real}$. Simply speaking, the smaller was the difference between $R_{real}$ and the estimated measurement noise variance, the higher was the estimation accuracy.

Table 5 shows that DAE estimated the measurement noise variance most accurately, except when the added noise was 40% of the original standard deviation σ. However, these results were somewhat different from those of the Kalman filtering accuracy in Table 2. For example, when the sequence length was 512 and the standard deviation of the noise was 40% of σ, DAE was the best in the filtering accuracy, as shown in Table 2, but WT was the best in the measurement noise variance estimation, as shown in Table 5. These two accuracy results are different due to the other parameters of Kalman filtering in addition to the measurement noise variance, i.e., not only the measurement noise variance but also other input parameters such as the process noise variance and transition matrices greatly affect the filtering accuracy of the Kalman filter. Therefore, if these values were appropriately adjusted to the sensor data, the filtering performance would also be equal or similar to the noise variance estimation result.

Table 6 shows the measurement noise variance estimation result on the voltage data. As shown in the table, $R_{WT}$ was the most similar to $R_{real}$ when the standard deviation of the noise was 20% and 40% of σ, while $R_{DAE}$ was the most similar to $R_{real}$ when the standard deviation of the noise was 60%, 80%, and 100% of σ. As in Table 2 and Table 5, the estimation accuracy of the measurement noise variance was somewhat different from the filtering accuracy in Table 3, and this difference might also result from the other parameters used in Kalman filtering, as described above.

Through the experimental results in Section 5.2 and Section 5.3, it was confirmed that the proposed noise variance estimation approach worked correctly and efficiently. In particular, the experimental results presented in Section 5.3 show that the proposed methods WT and DAE estimated the measurement noise variance accurately, improving the filtering performance when compared to the existing method EXP. Thus, it can be concluded that both the transform- and learning-based methods proposed in this paper are useful for recommending the measurement noise variance in Kalman filtering. More specifically, WT and DAE provide a high performance compared to EXP; WT has an advantage of being easy to use; and DAE has an advantage of requiring no additional parameters such as *f* in WT.

From the perspective of ordinary users, they may use either of the proposed methods to estimate the measurement noise variance in streaming environments where data are constantly being generated. However, if few data are available, then WT is more preferable than DAE since DAE generally needs many data for training and to obtain a better performance. Ordinary users might have some difficulty in constructing a denoising autoencoder model. However, there are many easy-to-use machine learning libraries such as TensorFlow (https://www.tensorflow.org/), PyTorch (https://pytorch.org/), and Keras (https://keras.io/) that provide many built-in models for various applications and ordinary users may simply use a pre-trained model such as the one presented in this section, still obtaining a relatively good performance for their application.

## 6. Conclusions

This paper proposes a novel method of estimating the measurement noise variance, an important parameter for efficient Kalman filtering. In Kalman filtering, the users need to give the measurement noise variance as an input parameter to the Kalman filter, but ordinary users have difficulty in acquiring the detailed noise information from the complicated sensor data. Thus, they often determine the measurement noise variance experientially or arbitrarily and use it in Kalman filtering, resulting in a poor filtering performance. To solve this problem of the experiential approach, this paper presents an analytical approach for estimating the measurement noise variance from the input sensor data. The basic idea is to estimate the original data from the input sensor data, calculate the noise variance from these estimated original data and input sensor data, and use the variance as the measurement noise variance. For this, first, an overall working framework of the analytical approach is presented and then transform- and learning-based methods are proposed to estimate the original data and calculate the measurement noise variance. The transform-based method uses a wavelet transform and the learning-based method uses a denoising autoencoder, which is a special type of neural networks. Experimental results on real power and voltage data show that the proposed analytical methods estimated the measurement noise variance accurately, improving the accuracy of Kalman filtering compared to the experiential method.

There are several research directions for future work. First, although the proposed methods are general enough to be useful in other contexts, a further investigation is needed to see if they are also applicable to the multivariate or extended Kalman filter. Second, more quantitative comparisons with other estimation methods are required to further validate the effectiveness and efficiency of the proposed methods. Another interesting direction for future work is to consider other noise environments such as colored noise or noise with non-Gaussian distributions to see if the proposed methods are robust and flexible enough for various noise environments. It would also be interesting to conduct further experiments on other real sensor data such as for IoT. Lastly, it is planned to further optimize the denoising autoencoder model by considering other parameters of Kalman filtering.

## Figures and Tables

**Table 1:** Hyperparameters used for training the denoising autoencoder.

Parameter	Value	Remark
Learning rate	0.01	A value which determines how much the weights of the model are adjusted.
Batch size	200	The number of training sequences used in each iteration.
Optimizer	Adam	An algorithm used to update the weights of the model.
# of epochs	500	An epoch is one complete presentation of the entire training data to the model.

**Table 2:** Filtering accuracy on power data of different sequence lengths. The boldface numbers denote the best value in each experiment.

Noise	Sequence Length = 512			Sequence Length = 1024			Sequence Length = 2048		
	EXP	WT	DAE	EXP	WT	DAE	EXP	WT	DAE
20%	871.7	368.0	**343.4**	869.5	367.6	**342.5**	866.5	366.3	**341.8**
40%	874.4	486.1	**484.8**	874.6	485.1	**484.0**	875.6	484.4	**483.6**
60%	875.7	**575.3**	580.5	879.0	**574.4**	579.7	882.3	**573.9**	579.4
80%	887.1	**646.3**	653.5	884.1	**645.7**	652.9	883.1	**645.2**	652.6
100%	894.3	**704.5**	712.2	895.5	**704.0**	711.7	893.7	**703.6**	711.4

**Table 3:** Filtering accuracy on voltage data of different sequence lengths. The boldface numbers denote the best value in each experiment.

Noise	Sequence Length = 512			Sequence Length = 1024			Sequence Length = 2048		
	EXP	WT	DAE	EXP	WT	DAE	EXP	WT	DAE
20%	2391	**1527**	1642	2390	**1526**	1599	2378	**1524**	1585
40%	2395	**1707**	1777	2395	**1707**	1749	2386	**1705**	1740
60%	2407	**1823**	1873	2408	**1823**	1853	2408	**1821**	1846
80%	2416	**1908**	1946	2408	**1907**	1930	2414	**1906**	1924
100%	2428	**1981**	2012	2422	**1983**	1999	2422	**1979**	1994

**Table 4:** Average computation time (ms) for the measurement noise variance estimation.

Sequence Length	Power Data		Voltage Data	
	WT	DAE	WT	DAE
512	9.32	2.14	9.46	2.20
1024	18.68	2.58	19.17	2.80
2048	37.25	3.64	36.08	3.71

**Table 5:** Measurement noise variance accuracy on power data of different sequence lengths. The boldface numbers denote the best value in each experiment.

Noise	Sequence Length = 512			Sequence Length = 1024			Sequence Length = 2048		
	*R_real_*	*R_WT_*	*R_DAE_*	*R_real_*	*R_WT_*	*R_DAE_*	*R_real_*	*R_WT_*	*R_DAE_*
20%	0.045	0.071	**0.050**	0.045	0.071	**0.050**	0.045	0.070	**0.050**
40%	0.179	**0.172**	0.169	0.179	**0.171**	0.169	0.179	**0.171**	0.169
60%	0.402	0.340	**0.373**	0.402	0.340	**0.373**	0.402	0.339	**0.373**
80%	0.715	0.575	**0.662**	0.715	0.574	**0.662**	0.715	0.574	**0.664**
100%	1.118	0.876	**1.039**	1.118	0.876	**1.040**	1.118	0.876	**1.041**

**Table 6:** Measurement noise variance accuracy on voltage data of different sequence lengths. The boldface numbers denote the best value in each experiment.

Noise	Sequence Length = 512			Sequence Length = 1024			Sequence Length = 2048		
	*R_real_*	*R_WT_*	*R_DAE_*	*R_real_*	*R_WT_*	*R_DAE_*	*R_real_*	*R_WT_*	*R_DAE_*
20%	0.420	**0.502**	1.661	0.420	**0.497**	1.166	0.420	**0.495**	1.030
40%	1.680	**1.446**	2.613	1.680	**1.442**	2.118	1.680	**1.439**	1.983
60%	3.780	3.018	**4.185**	3.780	3.014	**3.694**	3.780	3.012	**3.560**
80%	6.719	5.227	**6.390**	6.719	5.223	**5.905**	6.719	5.223	**5.769**
100%	10.50	8.059	**9.186**	10.50	8.057	**8.708**	10.50	8.058	**8.580**
